# Letter to the Editor: A Few Suggestions for Preventing Failure of Ultrasound-Guided Blocks for Below the Shoulder Surgery

**DOI:** 10.5041/RMMJ.10286

**Published:** 2017-01-30

**Authors:** Abhijit Nair

**Affiliations:** Department of Anesthesiology, Basavatarakam Indo-American Cancer Hospital & Research Institute, Hyderabad, Telangana State, India

**To the Editor,**

We read with great interest the original research published by Anatoli Stav et al.^1^ in which they compared supraclavicular (SCL), infraclavicular (ICL), and axillary (AX) approaches to the brachial plexus with ultrasonography for upper limb surgeries.^1^ They concluded that all approaches can be used to provide adequate anesthesia for upper limb surgeries below the shoulder. Nevertheless, they also experienced some sparing and failed blocks: 3 patients from the SCL group, 3 from the ICL group, and 4 from the AX group had a positive pin-prick test; 2 patients from the AX group received sedation supplementation; and 1 patient in the AX group experienced ulnar sparing which required a general anesthetic. Of the 13 failed blocks, 7 (i.e. ~50%) utilized the axillary approach to the brachial plexus. In this letter, we have tried to analyze and provide an explanation regarding the possible causes of the failed blocks performed under ultrasound guidance, particularly for the axillary approach.

In the SCL approach, the target structures are trunks arranged in a bundle-like sheath, which resemble a cluster of grapes on ultrasonography. Therefore, after visualizing the plexus, which is usually posterolateral to the pulsating subclavian artery, a single injection is usually enough to anesthetize the trunks in their entirety. However, the ulnar nerve is usually spared as the inferior trunk is located medially. A medially directed needle is not recommended as there is a high risk of pneumothorax due to inadvertent puncture of the pleura.^2^

The ICL approach targets the cords of the brachial plexus, located around the second part of the axillary artery. This is the best approach for elbow surgeries. The medial, lateral, and posterior cords of the brachial plexus are encountered with this approach, and, as the name suggests, the cords are named according to their relation to the second part of the axillary artery. This approach is best suited for catheter placement for continuous analgesia.^3^

The AX approach is ideal for hand surgeries and has the least complications. Musculocutaneous nerve sparing is the most common problem with the AX block, particularly when using nerve stimulation and landmark techniques.^4^ The musculocutaneous nerve arises from the lateral cord of the brachial plexus, pierces the coracobrachialis muscle, and travels to the forearm to continue as the lateral cutaneous nerve of the forearm. From there it innervates the lateral side of the forearm.^5^ Stav et al. referred readers to Vander Beek’s axillary brachial plexus block from *The Neuraxiom Playbook of 9 Essential Blocks: A Handbook of Ultrasound Guided Regional Nerve Blocks*,^6^ but they have not explained whether or not they separately blocked the musculocutaneous nerve with the AX approach. On ultrasonography, the musculocutaneous nerve appears as an oval to flat-oval to triangular structure located in the substance of the coracobrachialis muscle. However, anatomic variations are also described where the nerve is seen outside the coracobrachialis muscle.^7^ Therefore identifying the nerve and blocking it correctly is important, especially for hand surgeries utilizing an AX approach.

The median, ulnar, radial, and musculocutaneous nerves are the terminal nerves of the brachial plexus; all of them should be blocked for adequate upper limb anesthesia, especially when using ultrasound to perform the block. In case of sparing, the nerves (radial, ulnar, and median) can be blocked separately at the elbow level using a high-frequency ultrasound probe.

We agree with the authors that the SCL, ICL, or AX approaches to the brachial plexus can be used for surgery of the upper extremities below the shoulder. We also agree with the comment that an additional block of the intercosto-brachial and medial brachial cutaneous nerves is important in all cases. However, for a 100% successful block, the anesthesiologist should block the ulnar nerve separately using the SCL approach and the musculocutaneous nerve with the AX approach. After an ICL approach, if any sparing is noted in the distribution of a nerve, it should be blocked separately at the level of the elbow.

## Abbreviations

AX: axillary
ICL: infraclavicular
SCL: supraclavicular
